# Performing region-specific tasks does not improve lower extremity patient-reported outcome scores

**DOI:** 10.1186/s42836-024-00261-3

**Published:** 2024-07-07

**Authors:** Moritz J. Sharabianlou Korth, Wade A. Banta, Prerna Arora, Robin N. Kamal, Derek F. Amanatullah

**Affiliations:** 1 Department of Orthopaedic Surgery, Stanford Medicine, 450 Broadway Street, Redwood City, CA 94063 USA; 2 Department of Orthopaedic Surgery, University of Hawaii, 1356 Lusitana Street, Honolulu, HI 96813 USA

**Keywords:** THA, TKA, PROM

## Abstract

**Background:**

Patient-reported outcome measures quantify outcomes from patients’ perspective with validated instruments. QuickDASH (Quick Disability of Arm, Shoulder and Hand, an upper extremity PROM) scores improve after completing instrument tasks, suggesting patient-reported outcome results can be modified. We hypothesized that performing lower extremity tasks on the knee injury and osteoarthritis outcome score for joint reconstruction (KOOS-JR) and hip disability and osteoarthritis outcome score for joint reconstruction (HOOS-JR) instruments would similarly improve the scores.

**Methods:**

Forty seven hip and 62 knee osteoarthritis patients presenting to a suburban academic center outpatient osteoarthritis and joint replacement clinic were enrolled and randomized to an intervention or a control group. Inclusion criteria were age over 18 years and English competency. Patients completed a HOOS-JR or KOOS-JR instrument, completed tasks similar to those of the instrument (intervention) or the QuickDASH (control), and then repeated instruments again. Paired and unpaired t-tests were used to compare the intervention and control group scores before and after tasks.

**Results:**

There was no significant difference in total or individual scores after task completion compared to baseline in either the HOOS-JR or the KOOS-JR groups. There was no significant difference in the scores between the intervention or control groups.

**Conclusions:**

Disability may be less modifiable in the lower extremity than in the upper extremity, perhaps because upper extremity activities are more easily compensated by the contralateral limb, or because lower extremity activities are more frequent. Thorough evaluation of factors influencing patient-reported outcome measures is necessary before their extensive application to quality control and reimbursement models.

## Introduction

With the healthcare practice becoming patients-centered, patient-reported outcome measures (PROMs) are growing increasingly popular in clinical care, and popular instruments, such as the HOOS-JR and KOOS-JR, are frequently used for measuring outcomes after total joint arthroplasty [1, 2]. Using validated questionnaires, PROMs quantify clinical outcomes from a patient’s perspective and perception by converting symptoms into numerical scores. Patient-reported outcome measures track symptoms over time and provide insight into the biopsychosocial impact of medical conditions [3]. Patient-reported outcome measures are more and more used in orthopaedic surgery to assess and track patient outcomes, and while they are gaining support, important questions present themselves about how the data should be collected, visualized, shared, and used to improve the quality of care.

Before implementation, PROMs are thoroughly tested to evaluate validity, reliability, and responsiveness. However, these measurements do not always guarantee a PROM’s ability to accurately quantify disability. The tools used in PROMs are susceptible to cognitive biases, and psychological factors, such as anxiety and depression, which can influence scores [4]. Recent studies showed that even modifiable factors in the context in which the PROM was administered could change results. For example, scores on a region-specific PROM used in hand and upper extremity surgery (QuickDASH) could be improved by instructing patients to complete the functional tasks queried before completing the instrument [5]. The 11-item QuickDASH is a frequently used short-version of PROM designed to measure physical functions and symptoms of patients with injuries of the arm, shoulder, and hand [6].

While many orthopedic departments have started routinely collecting data by using PROMs in their outpatient clinics, standardized application of these instruments remains challenging [7]. Since standards regarding how specific PROMs should be administered can limit the contextual influence on the results, modifiable factors need to be identified. Therefore, we chose to investigate two joint-specific short-form PROMs frequently used in arthroplasty, the knee injury and osteoarthritis outcome score for joint reconstruction (KOOS-JR) and the hip disability and osteoarthritis outcome score for joint reconstruction (HOOS-JR) [1, 2]. HOOS-JR and KOOS-JR scores each range from 0 to 100, with 100 indicating no difficulty with tasks and 0 indicative of extreme limitation or inability to perform tasks [1, 2]. We hypothesized that patients completing the functional tasks queried on the HOOS-JR and KOOS-JR questionnaires would improve their scores, similar to the results seen after completing tasks queried on the QuickDASH [5]. We are not aware of studies that evaluate how simple actions, specifically completing functional tasks, affect region-specific PROMs of the lower limb. The purpose of this study was to investigate the external validity of commonly used PROM tools in the evaluation of lower extremity function. Since PROM’s are becoming more widely used in the evaluation of patients’ function and satisfaction with outcomes after lower extremity reconstruction, the validity of their results should be thoroughly investigated. Given that prior studies have questioned the persistence of upper extremity PROM results after completing functional tasks, we sought to evaluate the persistence of lower extremity PROM outcomes after functional tasks.

This study aimed to answer two questions: (1) Will H/KOOS-JR scores change after patients complete the tasks on the instrument compared with baseline scores? (2) Will the change in H/KOOS-JR score in an intervention (task-completion) group be different from that of a control group?

## Materials and methods

### Study design

After institutional review board approval, we enrolled patients from a suburban academic center outpatient osteoarthritis and joint replacement clinic. A research assistant approached all new and returning patients presenting to clinic for osteoarthritic knee or hip pain. Inclusion criteria were age older than 18 years and the ability to speak and read English. Patients meeting the inclusion criteria were approached after their visits with the physician and they provided informed consent. Patients were randomly assigned to an intervention or a control group. Sample size for hips and knees was determined by referring to the Quick-DASH used for hand exercise [5]. Patients were randomly assigned to an intervention or a control group. For the control group, we chose to use a set of activities in the upper extremity, reasoning that any differences in PROM outcome results of the lower extremity would not be likely to affect lower extremity pain response with activity and perception of function, and therefore would not impact PROM scores. The tasks performed were the same tasks that patients were asked to complete in the HOOS-JR and KOOS-JR PROM tools, with the exception of “walking on an uneven surface in the HOOS-JR tool.”

### HOOS-JR

#### Intervention group

Twenty-one patients were enrolled into the intervention group. All patients filled out the HOOS-JR instrument (baseline score) [5]. Patients’ demographics were collected in an additional questionnaire (Table 1). Patients completed tasks similar to the items listed on the HOOS-JR, including (1) rising from sitting; (2) bending to floor/picking up an object; (3) going up and down stairs; (4) lying down (turning over, maintaining hip position); (5) sitting. A follow-up HOOS-JR instrument was administered after tasks were completed.

**Table 1:** Demographics of patients enrolled in the HOOS JR and KOOS JR studies

Demographic factors	HOOS JR		KOOS JR	
	Intervention (*n* = 21)	Control (*n* = 26)	Intervention (*n* = 35)	Control (*n* = 27)
**Age** (years)	69.1 ± 10.2	65.9 ± 12.4	67.2 ± 8.9	62.7 ± 10.0
**Gender**				
*Male*	8 (38.1%)	15 (57.7%)	19 (54.3%)	15 (55.6%)
*Female*	13 (61.9%)	11 (42.3%)	16 (45.7%)	12 (44.4%)
**Annual household income**				
< *$49,999*	11 (52.4%)	7 (28.0%)	10 (30.3%)	8 (29.6%)
*$50,000–$99,999*	5 (23.8%)	5 (20.0%)	7 (21.2%)	5 (18.5%)
*$100,000–$149,999*	3 (14.3%)	4 (16.0%)	6 (18.2%)	3 (11.1%)
*$150,000–$199,999*	1 (4.8%)	2 (8.0%)	6 (18.2%)	3 (11.1%)
*$200,000–$249,999*	0 (0.0%)	2 (8.0%)	1 (3.0%)	4 (14.8%)
> *$250,000*	1 (4.8%)	5 (20.0%)	3 (9.1%)	4 (14.8%)
**Employment Status**				
*Full-time employed*	1 (4.8%)	9 (34.6%)	14 (40.0%)	10 (37.0%)
*Part-time employed*	4 (19.0%)	1 (3.8%)	2 (5.7%)	7 (25.9%)
*Retired*	11 (52.4%)	13 (50.0%)	14 (40%)	7 (25.9%)
*No work outside home*	0 (0.0%)	1 (3.8%)	0 (0.0%)	0 (0.0%)
*Disabled*	3 (14.3%)	1 (3.8%)	4 (11.4%)	1 (3.7%)
*Unemployed*	2 (9.5%)	0 (0.0%)	1 (2.9%)	2 (7.4%)
*Student*	0 (0.0%)	1 (3.8%)	0 (0.0%)	0 (0.0%)
**Highest level of education**				
*Elementary school*	0 (0.0%)	0 (0.0%)	1 (2.9%)	1 (3.7%)
*High school*	8 (40.0%)	4 (15.4%)	7 (20.6%)	5 (18.5%)
*2-year college*	4 (20.0%)	5 (19.2%)	7 (20.6%)	5 (18.5%)
*4-year college*	4 (20.0%)	3 (11.5%)	10 (29.4%)	8 (29.6%)
*Postgraduate degree*	4 (20.0%)	14 (53.8%)	9 (26.5%)	8 (29.6%)
**Relationship status**				
*Married*	8 (38.1%)	19 (73.1%)	17 (48.6%)	20 (74.1%)
*Domestic partnership*	1 (4.8%)	0 (0.0%)	4 (11.4%)	0 (0.0%)
*Single, never married*	3 (14.3%)	4 (15.4%)	3 (8.6%)	2 (7.4%)
*Single, divorced/separated*	6 (28.6%)	3 (11.5%)	5 (14.3%)	3 (11.1%)
*Single, widowed*	3 (14.3%)	0 (0.0%)	6 (17.1%)	2 (7.4%)
**Primary insurance**				
*Medicaid/Medi-Cal*	2 (14.3%)	0 (0.0%)	2 (7.4%)	3 (15.0%)
*Medicare*	9 (64.3%)	8 (50.0%)	15 (55.6%)	5 (25.0%)
*Military*	0 (0.0%)	0 (0.0%)	0 (0.0%)	0 (0.0%)
*Privately Insured*	3 (21.4%)	8 (50.0%)	10 (37.0%)	11 (55.0%)
*County Health Insurance*	0 (0.0%)	0 (0.0%)	0 (0.0%)	1 (5.0%)

#### Control group

Twenty-six patients were included in the control group. All patients filled out the HOOS-JR instrument (baseline score). Patients’ demographics were harvested in an additional questionnaire (Table 1). After completion of the baseline score, patients were asked to perform a set of upper extremity exercises similar to the tasks on the QuickDASH instrument, including (1) opening a tight jar of Play-Doh (2) simulating washing a clinic wall with a sponge for 20 s; (3) simulating washing one’s back with a sponge for 20 s; (4) using a knife to cut a piece of Play-Doh into four pieces. A follow-up HOOS-JR instrument was given after tasks were completed.

### KOOS-JR

#### Intervention group

The intervention group had thirty-five patients. All patients filled out the KOOS-JR instrument (baseline score). Patients’ demographics were collected in an additional questionnaire (Table 1). Patients completed tasks similar to the items listed on the KOOS-JR, including (1) straightening the knee fully; (2) rising from sitting; (3) twisting/pivoting the knee; (4) bending to floor/picking up an object; (5) going up or down stairs; (6) standing upright. A follow-up KOOS-JR instrument was administered after tasks were completed.

#### Control group

There were twenty-seven patients in the control group. All patients filled out the KOOS-JR instrument (baseline score). Patients’ demographics were taken in an additional questionnaire (Table 1). After completion of the baseline score, patients were asked to perform a set of upper extremity exercises similar to tasks on the QuickDASH instrument, including (1) opening a tight jar of Play-Doh (2) simulating washing a clinic wall with a sponge for 20 s; (3) simulating washing one’s back with a sponge for 20 s; (4) using a knife to cut a piece of Play-Doh into four pieces. A follow-up KOOS-JR instrument was administered after tasks were completed.

### Statistical analysis

Paired and unpaired *t*-tests were used to compare (1) PROM scores in the intervention group and control group after completing the tasks on the respective instrument compared to baseline; and (2) scores in the H/KOOS-JR total and individual components of the intervention group versus the control group. Continuous variables were reported as mean and standard deviation. Categorical variables were presented as number and percent. Statistical significance was set as a *P* value < 0.05.

## Results

For HOOS-JR, total scores (*P* = 0.388) as well as individual item scores did not change significantly after completion of the functional tasks in comparison to the baseline scores (Table 2). With KOOS-JR, total scores did not change significantly after completion of the functional tasks in comparison to the baseline scores *(P* = 0.171. Individual KOOS-JR item scores did not show any significant improvement, except for item 2 (Table 3, “twisting/pivoting your knee,” *P* = 0.041).

**Table 2:** Results of HOOS-JR instrument. Legend: Comparison of mean scores and standard deviation on the HOOS, JR instrument results before (baseline) and after (follow-up) completing either a list of tasks similar to the items in the HOOS, JR (intervention), or the QuickDASH (control) instrument

Group	Baseline	Follow-Up	*P* value
*(1) Going up and down stairs*			
Intervention	2.5 ± 1.1	2.3 ± 1.2	0.254
Control	2.4 ± 0.9	2.5 ± 0.9	0.385
*P* value	0.646	0.575	-
*(2) Walking on an uneven surface*			
Intervention	2.3 ± 1.0	2.1 ± 1.0	0.362
Control	2.3 ± 1.1	2.2 ± 1.0	0.343
*P* value	0.967	0.637	-
*(3) Rising from sitting*			
Intervention	2.0 ± 1.1	2.0 ± 1.0	0.500
Control	2.1 ± 1.1	2.0 ± 0.9	0.444
*P* value	0.927	0.974	-
*(4) Bending to floor/pick up an object*			
Intervention	2.2 ± 1.0	2.1 ± 1.1	0.389
Control	2.5 ± 0.9	2.4 ± 0.9	0.377
*P* value	0.342	0.324	-
*(5) Lying in bed (turning over, maintaining hip position)*			
Intervention	2.1 ± 0.9	2.0 ± 1.0	0.436
Control	2.3 ± 1.0	2.2 ± 1.0	0.342
*P* value	0.464	0.624	-
*(6) Sitting*			
Intervention	1.6 ± 0.9	1.7 ± 0.9	0.369
Control	1.4 ± 1.2	1.4 ± 1.0	0.500
*P* value	0.561	0.330	-
*(7)Total Score (0–24)*			
Intervention	12.2 ± 5.7	11.7 ± 5.9	0.388
Control	13.0 ± 5.1	12.7 ± 4.5	0.419
*P* value	0.615	0.504	-
*(8) Interval Score (0–100)*			
Intervention	49.9 ± 17.7	51.6 ± 19.2	0.380
Control	49.1 ± 16.3	50.2 ± 14.2	0.397
*P* value	0.870	0.767	-

**Table 3:** Results of KOOS JR instrument. Legend: Comparison of mean scores and standard deviation on the KOOS, JR instrument results before (baseline) and after (follow-up) completing either a list of tasks similar to the items in the KOOS, JR (intervention) or the QuickDASH (control) instrument

Group	Baseline	Follow-Up	*P* value
*(1) Knee stiffness after first wakening*			
Intervention	2.4 ± 1.1	2.4 ± 0.9	0.453
Control	2.3 ± 0.9	2.2 ± 1.0	0.444
*P* value	0.524	0.463	-
*(2) Twisting/pivoting on your knee*			
Intervention	2.9 ± 1.0	2.5 ± 1.1	0.041*
Control	2.5 ± 0.9	2.4 ± 0.9	0.332
*P* value	0.107	0.763	-
*(3) Straightening knee fully*			
Intervention	2.1 ± 1.0	1.8 ± 1.0	0.131
Control	2.3 ± 1.0	2.1 ± 1.1	0.303
*P* value	0.408	0.218	-
*(4) Going up or down stairs*			
Intervention	2.9 ± 1.0	2.9 ± 0.9	0.452
Control	2.7 ± 1.0	2.8 ± 0.9	0.278
*P* value	0.394	0.857	-
*(5) Standing upright*			
Intervention	2.0 ± 1.0	2.1 + 1.0	0.450
Control	1.9 ± 1.1	2.1 ± 1.2	0.235
*P* value	0.701	0.747	-
*(6) Rising from sitting*			
Intervention	2.7 ± 0.8	2.4 ± 0.8	0.093
Control	2.1 ± 0.8	2.4 ± 1.1	0.199
*P* value	0.014 *	0.904	-
*(7) Bending to floor/pick up an object*			
Intervention	2.6 ± 0.9	2.3 ± 1.0	0.089
Control	2.3 ± 1.1	2.3 ± 1.1	0.403
*P* value	0.201	0.857	-
*(8) Total score (0–28)*			
Intervention	17.1 ± 5.9	15.8 ± 5.7	0.171
Control	16.1 ± 5.6	16.4 ± 6.2	0.409
*P* value	0.484	0.672	-
*(9) Interval score (0–100)*			
Intervention	42.5 ± 14.1	46.3 ± 13.4	0.127
Control	46.4 ± 14.7	44.8 ± 17.8	0.358
*P* value	0.288	0.716	

^*^Statistically significant (*P* < 0.05)

There was no significant difference in the scores between the intervention group and the control group (Table 2) when taking the HOOS-JR. No significant difference was found in the scores between the intervention group and the controls, except for item 6 (Table 3, “rising from siting,” *P* = 0.014) when taking the KOOS-JR.

## Discussion

We found that completing lower extremity functional tasks had no effect on patient performance on the lower extremity-specific PROMs HOOS-JR and KOOS-JR when compared to baseline scores.

Similarly, we found that completion of functional tasks did not affect patient H/KOOS-JR score compared to controls who performed non-lower extremity-related tasks.

Cognitive biases and psychological factors have been found to influence outcomes, and recent studies have highlighted the importance of the context in which the PROMs are administered [4, 5]. It is reasonable to assume that modifiable factors, such as environment (i.e., waiting room, etc.), timing (pre-, or post-visit) and personnel (who is administering the questions) can influence PROM scores. Shapiro et al. showed that by asking patients to complete functional tasks as queried on the QuickDASH, reported disability decreased. [5]

Our findings showed that these effects were not seen with PROMs of the lower extremity. While prior studies have shown that results of the upper extremity PROM QuickDASH could be modified by completion of the activities described in the tool, lower extremity PROM modification with the activities has not been evaluated. The results of this study contribute to the validity of the use of the HOOS-JR and KOOS-JR after lower extremity disease. A possible explanation for the difference in our findings lies in the difference in PROM design and region tested. Functional tasks of the upper limb queried on the QuickDASH, for example, cutting and washing, allow for functional compensation using the contralateral side. Therefore, it is possible that patients might not have completed a queried functional task using their injured limb and may report disability based on an activity they perceive similar in character. In the intervention group, after completing the queried task, the patients might give a more accurate representation of their disability.

Unilateral functional impairment of the lower limb, as found by the commonly used region-specific PROMs (H/KOOS-JR), is not as easily compensated by the other leg. For example, walking stairs or getting out of a chair will almost always involve both hips and/or knees. Considering the possibility that region-specific PROMs involving the lower limb, in this case H/KOOS-JR, baseline scores may more accurately reflect a patient’s perceived disability.

There are several limitations to our study. It is possible that patients, while completing the follow-up questionnaire, remembered their initial response for the baseline score. After completion of the functional tasks and demographic survey, there were 10–15 min between the completion of the two PROM sessions. It is possible that the time interval in between was not long enough to eliminate the influence of the baseline score from the post-task score. Future studies should consider using longer time intervals, and potentially even administer baseline instruments and follow-up on different days. Patients gave baseline scores shortly after their visits with an orthopaedic surgeon. Discussing disabilities and examination of the hip and knee might have influenced baseline scores through recall bias. To counter the possibility of priming patient’s before collecting baseline scores, it would be helpful to hand out questionnaires when patients checked in at clinic and before their visit with the surgeon. This study evaluated only the response on PROMs at a single point in time. As such, it is unable to account for possible baseline reduction in function unrelated to lower extremity disease.

## Conclusion

PROMs have been increasingly employed in orthopaedic surgery. Traditional postoperative assessment revolves around objective measurements collected by health care providers. PROMs are powerful tools that can empower patients’ voice by quantifying patients’ perspectives and perceptions. However, we know that modifiable factors can influence PROMs and the way questionnaires are administered differs between surgeons and institutions [3–5]. PROMs vary in study design, anatomic region and disabilities measured [1–3, 6]. Our results showed that modifiable factors that influence PROM results varied between questionnaires and study regions and could not simply be translated between seemingly similar PROM tools. Before PROMs can be broadly implemented into reimbursement models and quality control, modifiable factors that influence PROMs need to be thoroughly analyzed and standardized, and pragmatic and efficient ways to collect PROMs must be developed.

## Data Availability

The datasets used and/or analyzed during the current study are available from the corresponding author on reasonable request.

## Abbreviations

PROM: Patient reported outcome measure
QuickDASH: Quick disability of arm, shoulder, and hand
KOOS-JR: Knee injury and osteoarthritis score for joint reconstruction
HOOS-JR: Hip injury and osteoarthritis score for joint reconstruction
THA: Total Hip Arthroplasty
TKA: Total Knee Arthroplasty
